# Acknowledgment to the Reviewers of *Diseases* in 2022

**DOI:** 10.3390/diseases11010013

**Published:** 2023-01-18

**Authors:** Diseases Editorial Office

**Affiliations:** MDPI AG, St. Alban-Anlage 66, 4052 Basel, Switzerland

High-quality academic publishing is built on rigorous peer review. *Diseases* was able to uphold its high standards for published papers due to the outstanding efforts of our reviewers. Thanks to the efforts of our reviewers in 2022, the median time to first decision was 22 days and the median time to publication was 46 days. Regardless of whether the articles they examined were ultimately published, the editors would like to express their appreciation and thank the following reviewers for the time and dedication that they have shown *Diseases*:
Abdelfattah M. SelimLuiza GuilhermeAbdolrazagh Hashemi ShahrakiLuke MantleAbdul Arif KhanLu-Sheng HsiehAbdullah DemirtaşMaarten Van CleeffAdriana M. CalvoMagdalena HurkaczAgata StanekMaher Abdel NabiAhmed S. I. AlyMahesh KasthuriAlexander D. BotvinkinMahi M. MohiuddinAlexander KorthausMarc PocardAlfred O. AnkrahMarc SturgillAman Ullah KhanMarco CascellaAmir HadiMaria BucovaAna Judith Perisé-BarriosMaria Grazia PizzaAnanya Tupaki-SreepurnaMaria Pina DoreAnca Oana DoceaMaria Teresa GuagnanoAndrea FerrignoMario CioceAndrew D. HaddowMark W. DouglasAndrzej PawlikMarleide Da Mota GomesAngela C. GarinisMasaaki ShimataniAngels EscorsellMassimiliano RenziAnna SzumilewiczMassimo IacovielloAnna VilellaMatteo RiccòAnroop B. NairMatthaios P. KavvalakisAnu Anna GeorgeMauro GiacomelliAnudishi TyagiMaxim L. BychkovAo ZhangMaxime PichonArif SiddiquiMaxine A. WhittakerAsif KhaliqMaya MascarenhasAurelio Luna MaldonadoMehmet Ali ErgünBarbara SteccaMelanie BoeckmannBernhard RyffelMichael Rutledge DeBaunBin RenMichela BulloneBrett ToelleMiguel Angel OrtegaCălin CăinapMikhail BochkarevCaner ÇakiMingyi ChenCarla FeliceMirko DuradoniCarlo CampanaMirko WinklerCarmen Sílvia Valente BarbasMohamed SolimanCassie S. MitchellMohammad Amin MoosaviCatherine A. GordonMohammed AkbarChan Kyung KimMohd ImranChi Chun WongMohsin MaqboolChia-Tung ShunMoises DiagoChi-Chun HoMonika OlechChien-Feng LiMrinmoy GhoshChien-yu LinMuhammad IkramChunying LiMumtaz AnwarDan Cacsire Castillo-TongNathan Kumasenu MensahDaša StupicaNaveed Ullah KhanDavid ChambersNeeraj JainDawid SigorskiNeveen SaidDa-Yuan ChenNicola DecaroDemba SarrNicolae GicaDenes TothNicolas GendronDerek G ShendellNicoletta De SantisDiana M. LindquistNidia Aréchiga CeballosDiane MègeNiels QvistDidier G LeiboviciNikola M. PuvaĉaDihogo De MatosNina TumosaDmytro DmytriievNobumichi KobayashiDomenico De BerardisOleg S. GlotovDominik DłuskiOliver HaydenEdgar Tavares-da-SilvaPamela MaherEduarda MendesPankaj AhluwaliaElena Maria ElliPavel FialaElisavet StavropoulouPeriasamy SelvarajElsa VitalePhilipp SchreinerEmma EvergrenPietro ScicchitanoEnrique Romero-VelardePio Maria FurneriEstéfani García-RíosPravin KaldhoneEugenio RagazziQishi SongFaez KhanRachy AbrahamFan ZhuRafaella Sayuri IoshinoFerdinand Von EggelingRafal DankowskiFerhunde AysinRajeev SinglaFernando GõmezRajiv SahFrancesca MeliniRamasare A. PrasadFrancesco MassariRamona D’AmicoFrancesco ValituttiRenhau LiGabriele C. GhisleniRita MachaalaniGagandeep SagguRitu RavalGeorge LazaroiuRobert BosGeorge T. KoliopoulosRobert D. MorrisGernot RiedelRobert Steven EsworthyGian Luigi RussoRoberta IbbaGilberto Castañeda-HernándezRoberto ColasantiGiovanni ChiariniRongjin SunGiovanni PalleschiRosalba SiracusaGitana Maria AcetoRose-Marie SatherleyGiulio RossiRuei-Nian LiGiuseppe Della PepaRwik SenGrazyna GosciniakSamia ElseginyGrisha PirianovSandra BarbalhoGualtiero AlvisiSaurabh ChattopadhyayGulzhanat AimagambetovaSeamus O’ReillyGuomao ZhengSergio NavarroGuorong LiSeyed Ahmad Seyed AlinaghiHaigang WuShamik MajumdarHalil YildizShao-Fang NieHany Hamdy ArabSheng-Fan WangHarald W. PlattaShinji TakamatsuHassan MshindaShula ShazmanHerbert Júnior DiasSi ChenHideaki OkajimaSiaw BoonHong DuanSidharth MuralidharanHsien-Yuan LaneSilvia Yumi BandoHui-Hua HsiaoSnjezana PetrovicHung-Yi ChuangSo Young YooI-Chien WuSoraia MeloIlaria Tocco TussardiSpiros ParamythiotisIoannis KosmasStanley W. AshleyIsabella LeoStefan D. MomčilovićItamar GonçalvesSubhadip MukhopadhyayJacques CabaretSudip ShethJaideep MahendraSurya KantJajah FachirohSusana FrancoJan Pieter KonsmanSvetlana MerenkovaJana KoscovaTakayuki MasakiJean François DohertyTakeshi KannoJeremy WoodwardTatu RojalinJihan XiaThirumaran ThanabaluJingan LiThomas HegyiJoanna DąbrowskaTimothy LysykJoanna KaminskaTimothy N. C WellsJoaquim CarrerasTimotius Ivan HariyantoJochen SchneiderTomasz FrączykJoel LexchinTorbjörn LedinJohn BartkowskiTurki TurkiJon OdlandValéria Lima CarvalhoJonathan N. FlakValeria TrivelloneJosé Cleiton Sousa Dos SantosVasilios PergialiotisJoshy GeorgeVenerando RapisardaJulian MuñozVera SimoesJuliane FriemelVíctor M. RiveraJulio Plaza DíazW. Bijlsma MerijnJumpei SaitoWael El-DeebKai Siang ChanWalter R. SchummKatarzyna Kiec-KononowiczWanich SuksatanKatsushi TakebayashiWilber SabiitiKeiko HosohataWilliam PérezKhac-Minh ThaiWilliam SchwanKlavdia Oleschko LutkovaWilma BarcelliniKrzysztof TupikowskiWojciech ZygnerKun-Yi HsinWolter OosterhuisKurt LushingtonXiang WangLaura AlagnaXiaoxiao SunLaura CotoiXingshun QiLaura Ferrer-FontYashdeep PhanseLaura Mora-LópezYasushi ShibataLauro Velazquez-SalinasYasushi TsujiLea SpindlerYih Yuan ChenLianet MonzoteYinan JiangLidia Szulc-DąbrowskaYolandy LemmerLiqing ZangYonghong ZhangLuca PaduaYoung-Seoub HongLuca RevelliYves BorbélyLucia TerzuoliZdeněk KejíkLuis RamírezZygmunt Warzecha

